# Primary brachial artery aneurysm with associated basilic vein aneurysm

**DOI:** 10.1093/jscr/rjab056

**Published:** 2021-03-29

**Authors:** Khaleel A Hamdulay, Peter E Laws, Carmen M Ruiz

**Affiliations:** 1 Department of Vascular, Endovascular and Transplant Surgery, Christchurch Hospital, Canterbury District Health Board, Christchurch, New Zealand; 2 Department of Vascular Surgery, Nelson Hospital, Nelson Marlborough District Health Board, Nelson, New Zealand

## Abstract

There are but a handful of reported brachial artery aneurysms, the majority of which are pseudoaneurysms or false aneurysms caused by trauma or fistula creation. True or primary brachial artery aneurysms are even more rare, and if they occur, they often do so in isolation. In this case report, we discuss the interesting finding of a large primary brachial aneurysm together with an adjacent aneurysmal basilic vein identified intra-operatively. This presentation was 21 years after the renal transplant and ligation of an arteriovenous fistula in that same arm. It is noteworthy that the fistula was in the forearm and far away from the site of the untouched brachial area.

## INTRODUCTION

The majority of peripheral artery aneurysms are of the popliteal artery, making up around 85% of these cases [1]. By contrast, brachial artery aneurysms account for a much smaller proportion of peripheral aneurysms and are therefore rare [2]. The most common causes are trauma, a result of infection, or iatrogenic causes, for example, following puncture for an endovascular procedure. These are all secondary causes—primary or true brachial aneurysms are even more rare, with some literature reporting 0.17% of peripheral aneurysms [3]. Furthermore, there is no documentation of the associated venous aneurysms. True brachial aneurysms are usually discovered incidentally, by presentation with a pulsatile or enlarging mass, with compressive symptoms on the adjacent structures such as nerves or with complications of an ischaemic limb due to a thromboembolic event [4, 5].

There have been a few cases in the literature of brachial artery aneurysm after the ligation of a previously functioning fistula [6]. It is known that pseudoaneurysms can also develop from a functioning fistula due to repeated haemodialysis access puncture. Our case report discusses a patient who presented with a true brachial aneurysm, but of particular interest, is the delay between the fistula ligation and presentation. We also focus on some of the challenges in identifying the best surgical management due to intra-operative findings such as the surprising discovery of the basilic vein aneurysm. Consent was obtained from the patient to publish the case details and images.

## CASE REPORT

We discuss a 69–year-old male who presented with a mass of the left arm. He was worked up for a musculoskeletal cause using magnetic resonance imaging (MRI) and was found to have a brachial artery aneurysm. A referral then followed to vascular surgery for the management of this uncommon finding.

His background included atrial fibrillation (AF) for which he was anticoagulated with warfarin, cadaveric renal transplant in 1998, with immunosuppression consisting of azathioprine, prednisone and ciclosporin, previous fistula, gout, stroke, radical prostatectomy with artificial urinary sphincter and recent legionella pneumonia. It remained pain-free until a throbbing ache developed about 6 months after noticing the lump, but the pain soon settled. There was some tingling in the index finger, indicating median nerve compression. There was no history of trauma to the left arm or any surgery to the brachial artery. He did however have a left radiocephalic arteriovenous fistula prior to his renal transplant in 1998, at which point, the fistula was ligated at the wrist.

**Figure 1:**
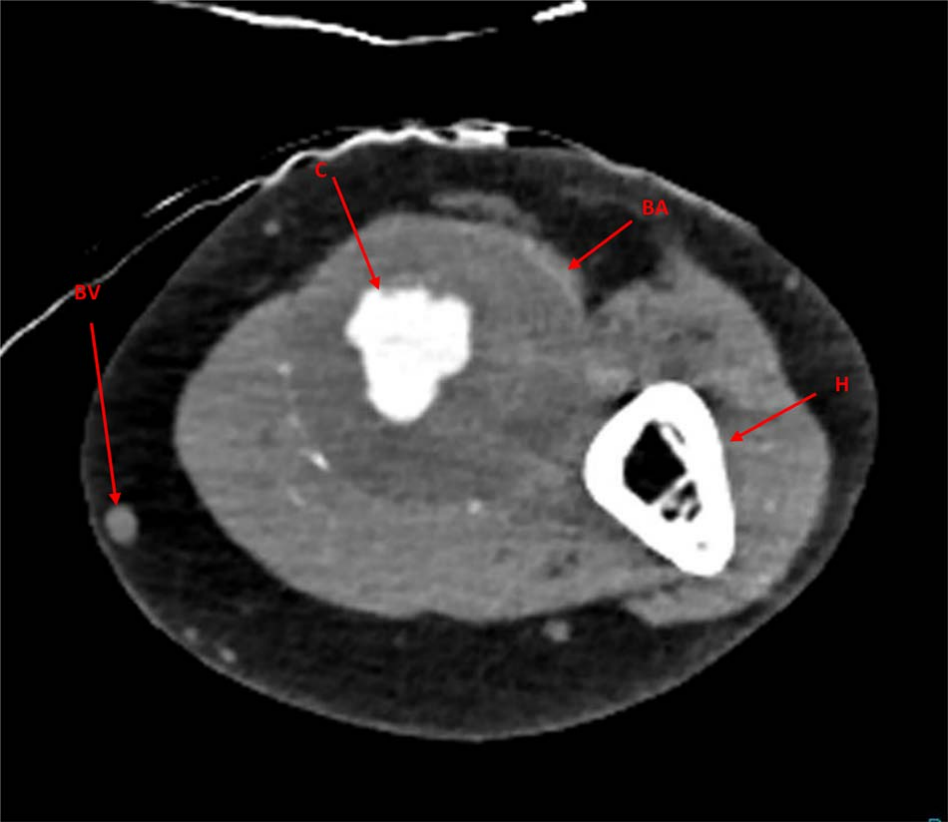
CTA imaging of brachial artery aneurysm of the left arm; BA, brachial aneurysm; BV, basilic vein; C, contrast through aneurysm lumen; H, humerus.

Examination revealed an expansive, pulsatile swelling over the whole upper arm. Ultrasound revealed the proximal axillary artery to be 10 mm, increasing to 14 mm below the axilla. The aneurysm measured at 45 mm diameter, decreasing to 16 mm above and 14 mm below the elbow. Significant thrombus was found within the aneurysm, but no distal embolization. The radial artery was small and the ulnar of normal calibre. A computated tomography angiogram (CTA) was arranged for surgical planning (Fig. 1).

**Figure 2:**
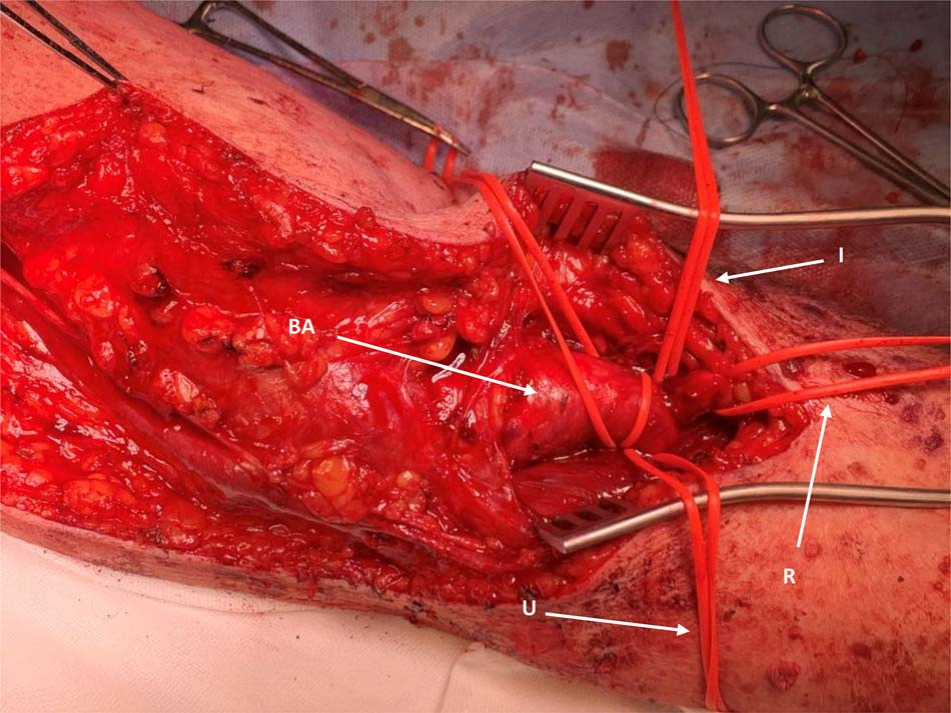
Distal operative site; the aneurysm is identified with branching vessels controlled with slings; BA, brachial aneurysm; R, radial artery controlled; U, ulnar artery controlled; I, interosseus controlled.

**Figure 3:**
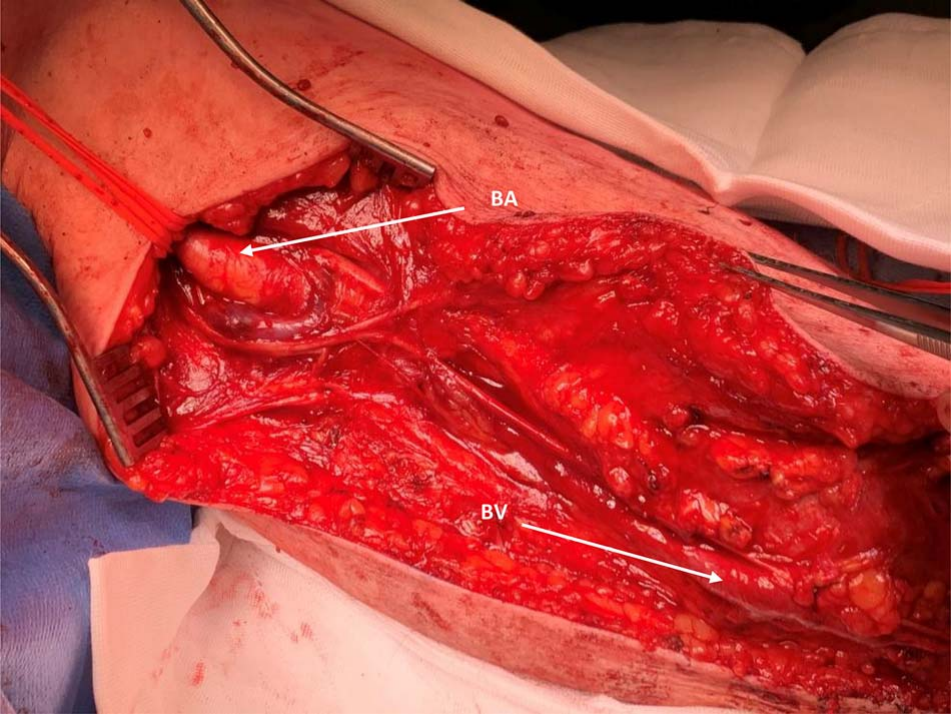
Proximal operative site; the aneurysmal proximal end of the brachial artery is seen (BA) and looped for control; BV, aneurysmal-associated basilic vein is seen and can be appreciated.

**Figure 4:**
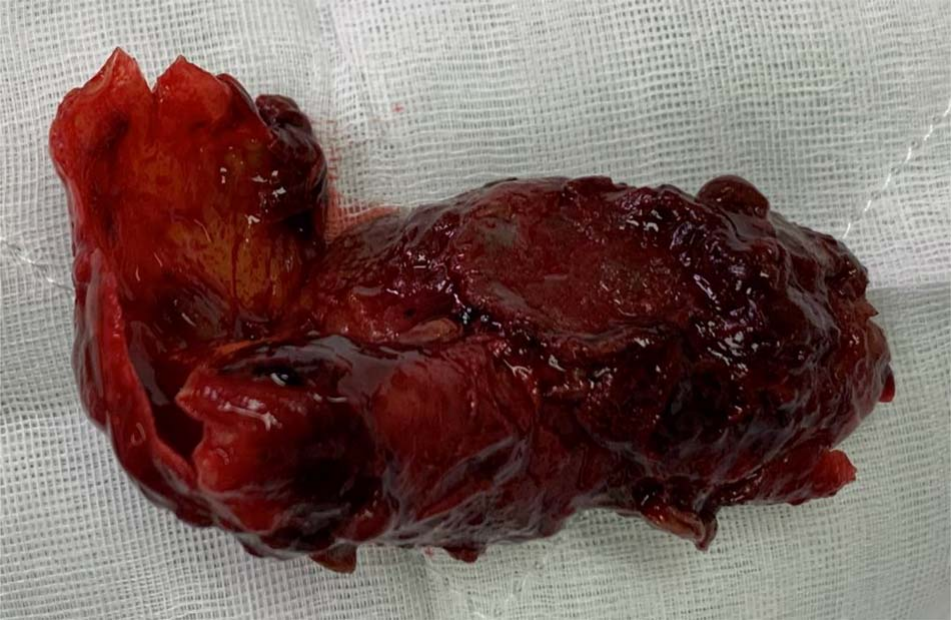
Thrombus resected from the aneurysm sac once opened.

**Figure 5:**
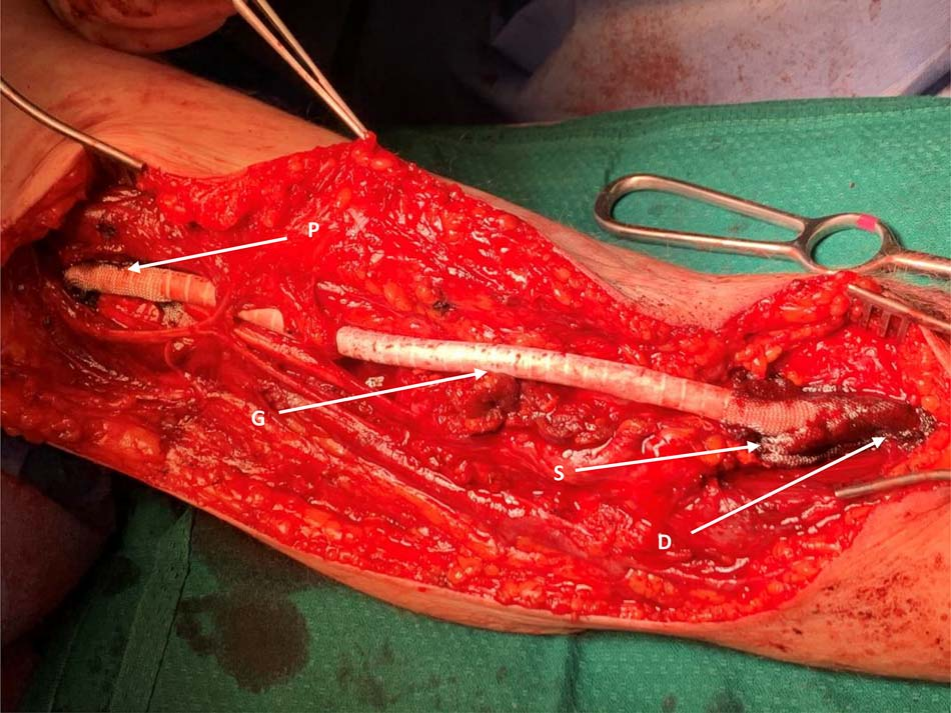
PTFE graft fashioned end-to-end as aneurysm replacement; G, graft made of PTFE; P, proximal end; D, distal end; S, surgicel applied to either end, intra-op for haemostasis.

Surgery proceeded with planned basilic vein harvest for use as an interposition graft. But on surgical exposure, the basilic vein was dilated, aneurysmal and clearly not suitable (Fig. 3). The decision was made for PTFE interposition graft instead. Distal control included identifying the aneurysm at the antecubital fossa with ulnar, radial and interosseus arteries controlled (Fig. 2). Proximally, the brachial artery was controlled. The aneurysm sac was opened and the thrombus was evacuated before being closed (Fig. 4). The PTFE conduit as an interposition graft was anastomosed (Fig. 5) with good pulsatile flow prior to closure.

## DISCUSSION

There are only a few cases documented of true or primary brachial artery aneurysms. Some of these cases discuss the formation of aneurysm after previous arteriovenous fistula ligation [6]. Of particular interest in our case is the 21-year interval between fistula ligation and presentation of the pulsatile mass. A recently published case describes a brachial aneurysm identified 15 years after fistula ligation [7]. It has been suggested that steroids play a role in aneurysm development as a result of tissue weakening and immunosuppression having a synergistic effect [8, 9]. Renal transplant patients will likely be on such medications, and it would therefore follow logically that they are at higher risk of these aneurysms forming. Our patient was indeed immunosuppressed on ciclosporin, azathioprine and prednisone for his transplant.

The unexpected intra-operative finding of the dilated basilic vein resulted in the PTFE graft being used instead. The associated aneurysmal basilic vein is of particular interest and has not been documented before. As most literature consists of case reports and case series, there are no randomized control trials (RCTs) about the management of these aneurysms. Best management is therefore not yet known. It is important to point out that our patient’s fistula prior to renal transplant was radiocephalic—at the wrist, and the brachial area was therefore untouched at the start of this operation. It has been suggested that fistula creation increases blood flow and produces shear forces in the vessel wall proximally, which could cause arterial dilatation as well as changes at the molecular level which compromise the internal elastic lamina [10].

It is important to document cases of such rare aneurysms as there are not enough data at present to guide the best management. Options suggested are open surgical replacement with vein or foreign artificial conduit versus endovascular covered stents. Ligation is another possibility, especially if the distal vessels are already thrombosed. Brachial aneurysms are also incredibly challenging to identify, and there are usually delays that occur during work up as in the case of our patient due to the suspicion of a musculoskeletal pathology. Nevertheless, our patient came to no harm and no thromboembolic events occurred.

## CONCLUSION

Brachial artery aneurysms are rare and true or primary aneurysms are much more so than pseudoaneurysms. They are difficult to diagnose and usually present with embolic complications. When identified, they should be surgically managed, but there may be unforeseen challenges such as the vein quality only being identified in the theatre. Surprisingly, we have identified an extended period between the arteriovenous fistula ligation and then the subsequent development of proximal aneurysm more than two decades later. We believe that more cases need reporting and data need to be captured to understand why this delayed development occurs.

## CONFLICT OF INTEREST STATEMENT

None declared.

## FUNDING

None.

## CONSENT

Consent was obtained from the patient to publish the case details and images.

## MEETING

Not presented at any previous meeting.
